# Impaired sharp-wave ripple coordination between the medial entorhinal cortex and hippocampal CA1 of knock-in model of Alzheimer’s disease

**DOI:** 10.3389/fnsys.2022.955178

**Published:** 2022-08-25

**Authors:** Tsukasa Funane, Heechul Jun, Stephanie Sutoko, Takaomi C. Saido, Akihiko Kandori, Kei M. Igarashi

**Affiliations:** ^1^Center for Exploratory Research, Hitachi, Ltd., Kokubunji, Japan; ^2^Department of Anatomy and Neurobiology, University of California, Irvine, Irvine, CA, United States; ^3^Laboratory for Proteolytic Neuroscience, RIKEN Center for Brain Science, Wako, Japan

**Keywords:** Alzheimer’s disease, hippocampus, entorhinal cortex, medial entorhinal cortex (MEC), sharp-wave ripples (SWRs)

## Abstract

Clinical evidence suggests that the entorhinal cortex is a primary brain area triggering memory impairments in Alzheimer’s disease (AD), but the underlying brain circuit mechanisms remain largely unclear. In healthy brains, sharp-wave ripples (SWRs) in the hippocampus and entorhinal cortex play a critical role in memory consolidation. We tested SWRs in the MEC layers 2/3 of awake amyloid precursor protein knock-in (APP-KI) mice, recorded simultaneously with SWRs in the hippocampal CA1. We found that MEC→CA1 coordination of SWRs, found previously in healthy brains, was disrupted in APP-KI mice even at a young age before the emergence of spatial memory impairments. Intriguingly, long-duration SWRs critical for memory consolidation were mildly diminished in CA1, although SWR density and amplitude remained intact. Our results point to SWR incoordination in the entorhinal-hippocampal circuit as an early network symptom that precedes memory impairment in AD.

## Introduction

Spatial memory impairments, including wandering and loss of spatial senses, are one of the most prominent symptoms in Alzheimer’s disease (AD) (Hope et al., 1994). Although past studies led to significant understanding of molecular and cellular mechanisms of AD, it remains unclear what type of activity impairment causes spatial memory impairments. Histological and functional imaging studies in AD patients showed that the entorhinal cortex, rather than the hippocampus, is the earliest brain region to have activity loss and atrophy in early phases of AD (Van Hoesen et al., 1991; Khan et al., 2014). Using *in vivo* electrophysiological recording from the amyloid precursor protein knock-in (APP-KI) mouse model, we previously showed that grid cells in the medial entorhinal cortex (MEC), critical for spatial navigation and memory (Moser et al., 2015), are impaired even at a young age before the emergence of spatial memory impairments (Jun et al., 2020). These evidence strongly suggests that the entorhinal cortex is the primary driver of pathophysiology in AD, but it remains largely unclear how the functional degradation of MEC causes spatial memory impairments in AD.

In healthy brains, sharp-wave ripples (SWR) are network activity underlying memory consolidation (Wilson and McNaughton, 1994; Foster and Wilson, 2006; Girardeau et al., 2009; Jadhav et al., 2012; Buzsaki, 2015). Long-duration SWRs especially play a critical role in consolidation, supposedly due to their higher spatial information content from longer concurrent spike replays and larger number of neurons involved in each SWR event (Fernández-Ruiz et al., 2019). Although the hippocampal CA3 critically contributes to the generation of SWRs propagating to CA1 (Buzsaki, 1986; Sullivan et al., 2011), SWRs in the layers 2/3 of the MEC also contributes to the generation of long and multiplex SWRs in CA1 (Yamamoto and Tonegawa, 2017). In AD brains, recent *in vivo* electrophysiological recording studies started to show disrupted SWRs in the hippocampus. Lower density (or abundance) of SWRs (the number of SWR events per time) was found in CA1 of 5xFAD mice (Iaccarino et al., 2016), apolipoprotein E4 mice (Gillespie et al., 2016; Jones et al., 2019), 3xTg AD mice (Benthem et al., 2020), and rTg4510 tau mice (Ciupek et al., 2015; Witton et al., 2016). A recent recording study from 5xFAD mice at the older age with memory impairments observed shorter SWR duration in CA1 (Prince et al., 2021). However, no previous studies tested SWRs in the entorhinal cortex in AD. We thus simultaneously recorded SWRs in the MEC and CA1 of APP-KI mice. To understand the role of entorhinal cortex in the pathogenesis of SWRs, we focused on recording from preclinical stage, that is, young age before the emergence of spatial memory impairments (Dubois et al., 2016; Sasaguri et al., 2017).

## Materials and methods

### Experimental design and the statistical tests used in the study

Data are shown with ± standard error. The animal numbers and sampled neuron numbers (biological replicates) were designed to achieve a power of more than 0.8. Both sexes of animals, randomized and blinded analyses were used (see Table 1). We initially tested difference between sexes and found no statistical difference, and thus combined data from sexes. For statistical testing, data were first tested for normal distribution using the Kolmogorov-Smirnov test (*p* < 0.05 cut-off). As distributions were not normal, we used Wilcoxon rank-sum test. Kolmogorov-Smirnov test was also used for comparing distribution.

**TABLE 1:** Sample information.

Genotype	Location	Sample number (Independent electrode number)
WT	CA1	48 (female 9, male 39)
WT	MEC	23 (female 5, male 18)
APP-KI	CA1	25 (female 5, male 20)
APP-KI	MEC	29 (female 7, male 22)

### Animals

Mice were maintained in standard housing conditions on a 12 h dark/light cycle with food and water provided *ad libitum*. As APP-KI mice have C57BL/6J background, we used C57BL/6J mice as control wild-type (WT) mice. Data were obtained from six WT mice and six APP knock-in mouse models (APP^*NL*–*G*–*F*^; Saito et al., 2014; Masuda et al., 2016) between 3 and 5 months of age. The electrical activity (local field potential, LFP) of neurons in the hippocampus and MEC were obtained. All procedures were conducted in accordance with the guidelines of the National Institutes of Health and approved by the Institutional Animal Care and Use Committee at the University of California, Irvine. Sixteen tetrodes with a total of 12 mice’s data, treated independently with records of effective neuronal activity, resulted in 125 independent data.

### Surgery and electrode preparation

**FIGURE 1:**
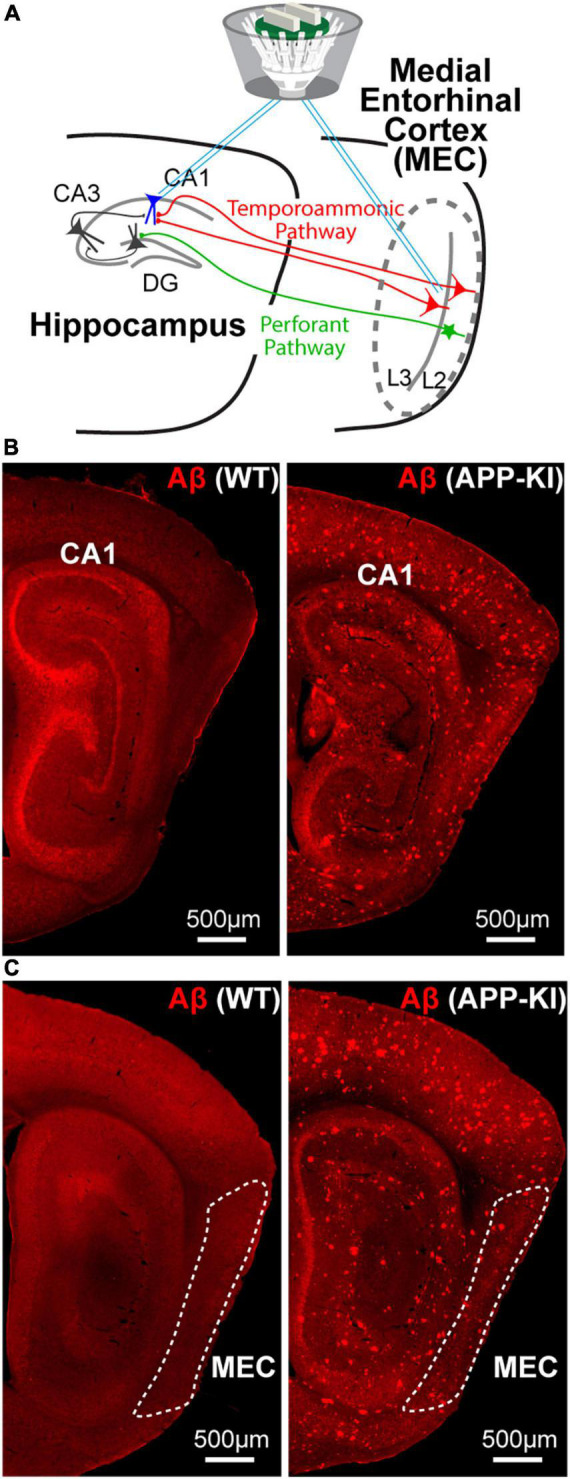
Simultaneous SWR recording from the MEC and hippocampus CA1 of young APP-KI mice. **(A)** A schematic of simultaneous SWR recording from medial entorhinal cortex (MEC) layer 2/3 and CA1 of the hippocampus. **(B,C)** Sagittal sections with anti-Aβ immunostaining for 3 mo WT mouse (left) and 3 mo APP-KI mouse (right).

All mice received a custom drive of 64 channels targeting CA1 or MEC (Figure 1A). In one group of animals, eight limbs targeted hippocampal CA1, and another eight limbs targeted the MEC. The tetrodes were constructed from 17 μm polyimide-coated platinum-iridium wires (90–10%) (California Fine Wire) twisted in four. A total of 16 tetrodes (i.e., 64 electrodes) were implanted in each mouse. The electrode tips were plated with gold and the electrode impedance was reduced to 150–300 kΩ at 1 kHz. Animals were anesthetized with isoflurane (air flow: 0.8–1.0 l/min, 1% isoflurane, adjusted for physiological conditions). The subject received buprenorphine subcutaneously at the start of the surgical operation. The depth of anesthesia was determined by examining the tail and pinch reflexes as well as by breathing. During induction of anesthesia, the animals were secured to a Kopf stereotactic frame. A craniotomy was performed in the hippocampus (approximately 1 × 1 mm) at 2.5 mm AP 2.5 mm ML and in the entorhinal cortex (approximately 1 × 1 mm) 0.2 to 0.4 mm anterior to the midline and 3.5 mm ML. The dura mater was carefully removed, and electrodes were implanted. A stainless-steel screw fixed to the skull in the upper cerebellum served as the ground electrode. The recording drive was secured to the skull using dental cement.

### Equipment and training procedures

**FIGURE 2:**
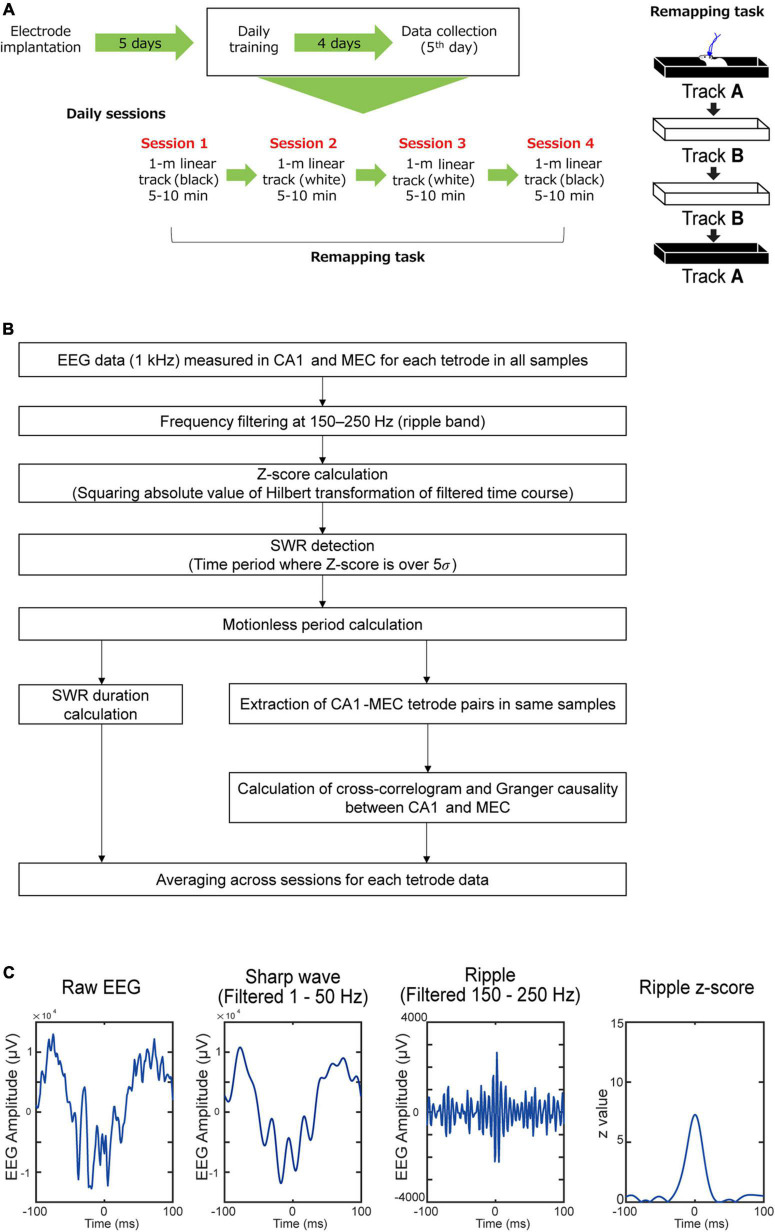
Experimental procedure. **(A)** *In vivo* electrophysiological recording were performed while APP-KI mice were running linear tracks. **(B)** Flow of the analysis used in the study. **(C)** An example SWR event defined by 150–250 Hz frequency band.

Behavioral training was initiated 5 days after surgery, and data collection was performed on day 5 of training (Figure 2). The task required that the mice run on two 1-m-long linear tracks. Track A is a black acrylic enclosure decorated with a pattern of white boards across the track and black rubber floors on the inner wall. Track B is a white acrylic enclosure with clear black boards on the inside walls of the track and white sandpaper floors. During the remapping task, animals were trained to run in a sequential order of track A → track B → track B → track A. The two tracks were in the same room. The animals were given a 5-min rest between the session. The run was motivated by placing a cookie piece at the end of the linear track. Each session lasted 5–10 min. On the linear tracks, the mice ran 10 full laps (back and forth).

### Histology and reconstruction of recording positions

Electrode positions were confirmed by anesthetizing the drive-implanted mice using isoflurane and performing small electrolytic lesions through passing current (10 μA for 20 s) through the electrodes. Immediately after this, the mice received an overdose of isoflurane and were perfused intracardially with saline followed by 4% freshly depolymerized paraformaldehyde in phosphate buffer (PFA). The brains were extracted and stored in the same fixative overnight. After overnight cryoprotection in phosphate buffer saline with 30% sucrose at 4°C, tissue samples were embedded in O.C.T. mounting medium and sagittal sections (40 μm) were cut and stained with cresyl violet. All tetrodes were identified, and the tip of each electrode was found by comparison with adjacent sections. Only data from tetrodes in the hippocampal CA1 or the layer 2/3 of dorsal MEC was collected for analysis. The electrical lesions often made tip holes in the brain section larger so that they span across cell layers. To ensure that the LFP was recorded from cell layers (either in pyramidal cell layer in CA1 or layers 2/3 in the MEC) we used LFP data only from tetrodes that had spiking activity of principal neurons for LFP analyses. Some of the MEC sessions were recorded at positions 40 or 80 μm above the terminal point of electrodes, which were estimated to be within layers 2/3 in the MEC. If these sessions had spikes distinct from previous and following sessions, they were included as distinct data sets.

### Data collection

Local field potentials (LFPs) were recorded in a single-ended fashion using a skull ground in the upper cerebellum. The tetrodes were multi-channel, impedance matched, and connected to a single gain head stage (Neuralynx). The output of the headstage was transmitted to the data acquisition system (Neuralynx). Unit activity was amplified 3,000–5,000-fold, bandpass filtered at 600–6,000 Hz. The LFP signal was recorded at a sampling rate of 2,000 Hz per tetrode at a frequency range from 0 to 475 Hz. Notch filters were not applied. The animal’s position recording system tracked the positions of the two light emitting diodes (LEDs) on the head stage (sampling rate 50 Hz) with an overhead video camera.

### Data analysis

Analyses were performed with custom MATLAB codes. The analysis flow used in the study is shown in detail in Figure 2. Each step of this flow is explained in the following sections. All data is shown as mean ± SE. All statistical testing assumed a non-parametric distribution and Wilcoxon rank sum test was used. We did not observe any statistical differences when data from males and females were compared, and thus combined them together.

### Detection of sharp-wave ripples

The EEG data were converted to discrete-time analytic signals by Hilbert transformation after applying a frequency filter at 150–250 Hz (Gillespie et al., 2016) as a ripple frequency domain. The power absolute value of the discrete-time analytic signal was then acquired as a *z*-value. When the *z*-value exceeded 5σ (σ is standard deviation) (Cheng and Frank, 2008; Jones et al., 2019), it was detected as an SWR and used for later analysis. Using mouse movements tracked by LEDs, SWRs were further restricted to the period at speeds less than 2 cm/s (immobile state).

### Sharp-wave ripples parameters calculation

#### Duration of the sharp-wave ripples

SWR duration was defined as the duration of EEG exceeding 5 times the standard deviation in *z*-values. SWR mean power (dB/Hz) was calculated by the mean of SWR power spectrum density (dB/Hz) among 150–250 Hz. The peak value of the SWR power spectrum was defined as the SWR frequency peak. SWR density (Hz) was calculated by the number of SWRs divided by total immobile-state duration. SWR density is the same as “SWR abundance” in some previous studies. This method defines SWR events using only ripple wave band width (150–250 Hz), and we further tested if sharp waves co-exist in these SWR events. Sharp wave events were independently defined as timepoints that exceeded 5σ in the EEG filtered at 1–50 Hz frequency band, and if a sharp wave event occurs within ±30 ms of a SWR event, it is considered as co-occurring (Maier et al., 2003). We found that 94.3 ± 1.6% of SWR events in the MEC and 96.9 ± 0.4% of SWR events in CA1 had co-occurring sharp waves in WT mice, and that 97.3 ± 0.6% of SWR events in the MEC and 96.1 ± 1.6% of SWR events in CA1 had co-occurring sharp waves in WT mice (Figure 2C).

### Cross-correlogram between sharp-wave ripples detected in hippocampus and entorhinal cortex

The coordination of SWRs between MEC and CA1 was assessed using cross-correlation method (Nowak and Bullier, 2000). Briefly, the number of SWRs in the MEC was counted -100 to +100 ms from the time of SWRs detected in CA1. The SWR counts were then normalized by total immobile-state duration.

### Granger causality analysis

To further assess coordination between SWRs between MEC and CA1, the Granger causality analysis was used (Granger, 1969). In the *z*-value time series of SWR, all *z*-values were kept for time bins that surpass 5σ, whereas values were set to 0 for other bins. Presence or absence of Granger causality (GC) between the *z*-value time series from CA1 and MEC was tested. All *z*-scores above 5σ regardless of velocity were used in case the SWR time-course data become very sparse for the Granger causality analysis. Results from Granger causality test (α = 0.05, false discovery rate correction for multiple correction),1 for causal and 0 for no causal, were averaged for four linear track sessions. This result was further averaged among all tetrode combinations. The group data of wildtype and AD models were compared by Wilcoxon rank sum test. For the Granger causality analysis, we used the MATLAB multivariate Granger causality (MVGC) Toolbox (Seth, 2010; Barnett and Seth, 2014). Akaike’s information criterion (AIC) and Bayesian information criterion (BIC) were used for vector autoregression model order optimization. The model order was optimized among 1–50.

## Results

### Sharp-wave ripples duration was specifically diminished in CA1 of amyloid precursor protein knock-in mice

**FIGURE 3:**
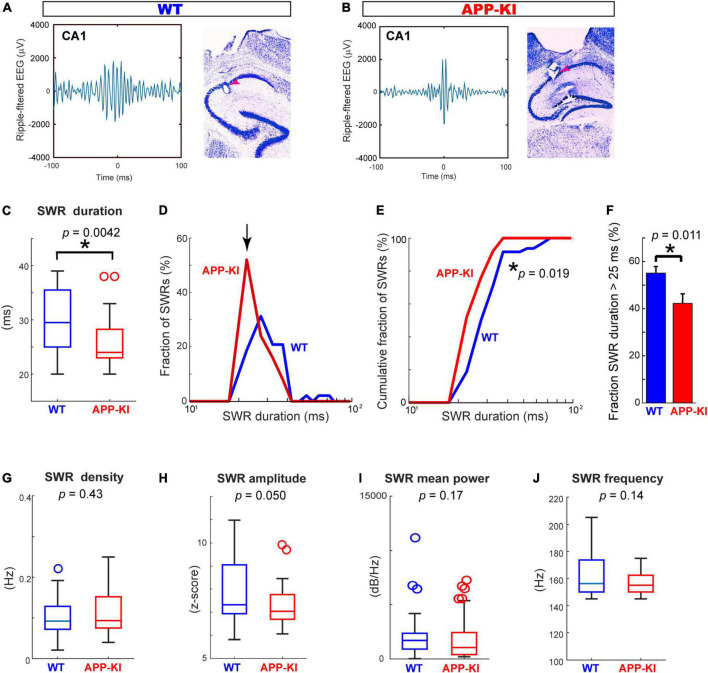
SWR duration was specifically diminished in CA1 of APP-KI mice. **(A,B)** Example SWR traces recorded from CA1 in WT **(A)** and APP-KI mice **(B)**. **(C)** SWR duration in CA1. *p* = 0.0042, Wilcoxon rank-sum test. **(D)** Fraction of SWRs plotted as a function of SWR duration. APP-KI mice showed larger fraction of SWRs with short duration in CA1 (arrow). **(E)** Cumulative fraction of CA1 SWRs as a function of SWR duration; *p* = 0.019, Kolmogorov-Smirnov test. **(F)** Fraction of CA1 SWRs with duration of >25 ms; *p* = 0.011, Wilcoxon rank-sum test. **(G–J)** SWR properties in CA1. SWR density **(G)**, SWR amplitude **(H)**, SWR mean power **(I)** and SWR Frequency peak **(J)**. *P*-values are from Wilcoxon rank-sum test. For all data in **(C–J)**, *n* = 48 and *n* = 25 CA1 recordings in WT and APP-KI mice, respectively. *p < 0.05.

We performed *in vivo* electrophysiological recording simultaneously from layers 2/3 of the MEC and hippocampal CA1 in APP-KI mice at 3–5 months of age (mo; *n* = 6 mice) (Figure 1A). APP-KI mice have Amyloid-β (Aβ) deposition in the CA1 and MEC at 3 mo (Figures 1B,C), whereas spatial memory impairment starts after 7 mo (Saito et al., 2014; Sasaguri et al., 2017; Jun et al., 2020). Control recording was performed from C57BL/6J mice (referred to as WT mice; *n* = 6 mice). Electroencephalography (EEG) data were obtained while mice ran 1-m linear tracks. We used two black and white linear tracks, where we recorded spike activities during remapping of place cells and grid cells (Jun et al., 2020). In this study, we analyze EEG activity obtained from these recordings, and data from two linear track sessions were averaged for each electrode. EEG was filtered at 150–250 Hz band, and SWRs were detected at time points where z-scored amplitude exceeded 5 times standard deviation (Gillespie et al., 2016; Figure 2). Data from *n* = 29 and *n* = 25 independent electrodes were collected from the MEC and CA1 of APP-KI mice, respectively. In WT mice, *n* = 23 and *n* = 48 electrodes were respectively implanted in the MEC and CA1. Recorded layers in the MEC and CA1 were subsequently validated histologically (Supplementary Figures 1, 2). Figures 3A,B show representative SWR events recorded from CA1 of WT and APP-KI mice. Because long-duration SWRs in CA1 are critically involved in memory consolidation (Fernández-Ruiz et al., 2019), we first assessed the duration of SWRs. We found that duration of SWRs recorded in CA1 of APP-KI mice was significantly shorter than that in WT mice (Figure 3C, 26.2 ± 1.0 ms in APP-KI mice vs. 32.1 ± 1.6 ms in WT mice; *p* = 0.0042, Wilcoxon rank sum test). A fraction plot of SWRs as a function of SWR duration shows that a large proportion of SWRs in APP-KI mice exhibited shorter duration than SWRs in WT mice (Figures 3D,E; *p* = 0.019, Kolmogorov-Smirnov test). SWRs with their duration of more than 25 ms were fewer in APP-KI mice (Figure 3F; *p* = 0.011, Wilcoxon rank sum test). By contrast, other properties of CA1 SWRs remained intact. In our ∼30-min recording sessions, we detected comparable density of SWRs between WT and APP-KI mice (Figure 3G, 0.097 ± 0.006 and 0.114 ± 0.012 SWR events/s from WT and APP-KI mice, respectively; *p* = 0.43, Wilcoxon rank-sum test). SWR amplitude (*p* = 0.050), mean power (*p* = 0.17) and frequency (*p* = 0.14) were also comparable (Wilcoxon rank-sum test, Figures 3H–J). These results raise an intriguing hypothesis that long-duration SWRs are specifically impaired in CA1 of APP-KI mice at the preclinical stage.

### Sharp-wave ripples characteristics in the medial entorhinal cortex were comparable between amyloid precursor protein knock-in mice and wild-type mice

**FIGURE 4:**
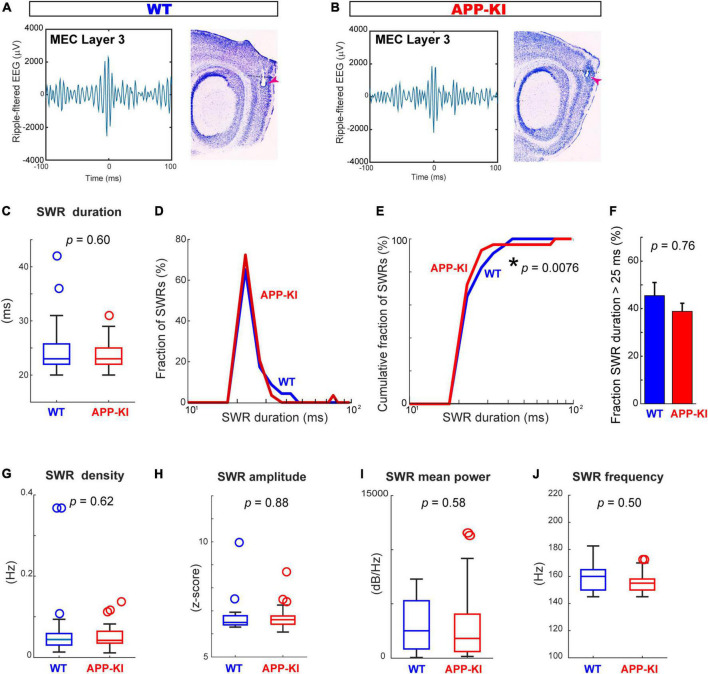
SWR characteristics in the MEC were comparable between APP-KI mice and WT mice. **(A,B)** Example SWR traces recorded from the MEC in WT **(A)** and APP-KI mice **(B)**. **(C)** SWR duration in MEC. *p* = 0.60, Wilcoxon rank-sum test. **(D)** Fraction of MEC SWRs plotted as a function of SWR duration. **(E)** Cumulative fraction of MEC SWRs as a function of SWR duration; *p* = 0.0076, Kolmogorov-Smirnov test. **(F)** Fraction of MEC SWRs with duration of > 25 ms; *p* = 0.76, Wilcoxon rank-sum test. **(G–J)** SWR properties in CA1. SWR density **(G)**, SWR amplitude **(H)**, SWR mean power **(I)** and SWR Frequency peak **(J)**. *P*-values are from Wilcoxon rank-sum test. For all data in **(C–J)**, *n* = 23 and *n* = 29 CA1 recordings in WT and APP-KI mice, respectively. *p < 0.05.

We next assessed properties of SWRs in the MEC (Figures 4A,B). In contrast to the diminished SWR duration in CA1, SWRs in the MEC showed comparable duration between APP-KI and WT mice (Figure 4C). While the distribution of SWR duration differs between APP-KI and WT mice (*p* = 0.0076, Kolmogorov-Smirnov test; Figures 4D,E), we also did not observe difference in the number of SWRs with duration >25 ms (Figure 4F). Furthermore, the density of SWR did not differ between WT and APP-KI mice (0.072 ± 0.019 and 0.053 ± 0.006 SWR events/s from WT and APP-KI mice, respectively; *p* = 0.62, Wilcoxon rank-sum test; Figure 4G). SWR amplitude (*p* = 0.88), mean power (*p* = 0.58) and frequency (*p* = 0.50) were all comparable between APP-KI and WT mice (Wilcoxon rank-sum test, Figures 4G–J). These data indicate that properties of individual SWR events remained unchanged in the MEC at preclinical stage, implying that generation mechanisms of SWR within the MEC are intact.

### Impaired sharp-wave ripples coordination between the medial entorhinal cortex and CA1

**FIGURE 5:**
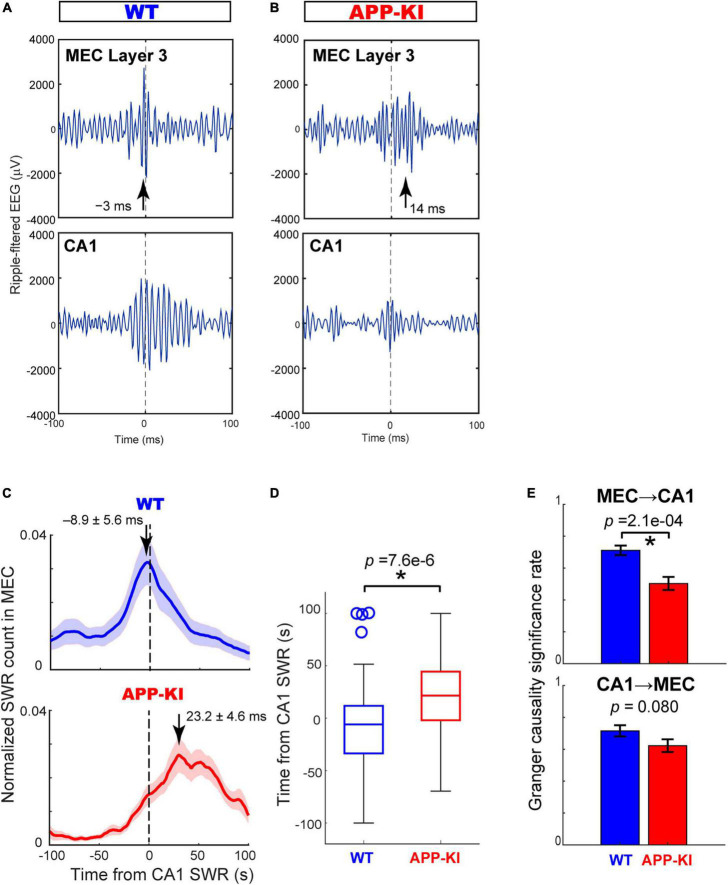
Cross-correlogram and Granger causality revealed impaired SWR coordination between MEC and CA1. **(A,B)** Example SWR traces occurring simultaneously in the MEC and CA1 in WT **(A)** and APP-KI mice **(B)**. SWRs are aligned at the amplitude peaks of SWRs in CA1. Arrows depict peak times of SWRs in the MEC. **(C)** Normalized counts of SWRs in the MEC triggered by SWRs in CA1 for WT mice (*n* = 65 electrode pairs) and APP-KI mice (*n* = 61 electrode pairs). **(D)** Peak time of MEC SWRs relative to SWRs occurring in CA1. *p* = 7.6 × 10^6^, Wilcoxon rank-sum test. **(E)** Significance rate obtained from Granger causality test on SWRs from MEC to CA1 (top, *p* = 2.1 × 10^4^, Wilcoxon rank-sum test) and from CA1 to MEC (bottom, *p* = 0.080, Wilcoxon rank-sum test). *p < 0.05.

The diminished duration of SWRs observed in CA1 raised an intriguing question as to whether the coordination of SWRs between the MEC and CA1, reported previously (Yamamoto and Tonegawa, 2017), were affected in APP-KI mice. We thus examined the coordination of SWR events between the MEC and CA1 using pairs of electrodes targeted in the two areas (*n* = 65 and *n* = 61 pairs from WT and APP-KI mice, respectively). Figures 5A,B show representative SWRs occurring simultaneously in the MEC and CA1. In healthy mice, SWRs in the MEC layers 2/3 are found to be preceding SWRs in the CA1 (Yamamoto and Tonegawa, 2017). However, the SWRs in the MEC of APP-KI mice appeared to be delayed from those in CA1 (Figure 5B). To assess the relative timing of SWRs between the MEC and CA1, we computed cross-correlograms of SWR counts recorded from the MEC around co-occurring SWRs in CA1 (Narayanan and Laubach, 2009; Figure 5C). WT mice showed SWRs in the MEC occurring 8.9 ± 5.6 ms prior to the SWRs in the CA1, consistent to the previous finding. By contrast, this advancement of SWRs in the MEC disappeared in APP-KI mice, with a peak of SWRs in MEC occurring 23.2 ± 4.6 ms *after* SWRs in the CA1 (*p* = 7.6 × 10^–6^, Wilcoxon rank-sum test; Figures 5C,D). To further test the idea that the MEC→CA1 SWR directionality is affected in APP-KI mice, we performed Granger causality analysis of SWR events for both MEC→CA1 and CA1→MEC directions (Granger, 1969). We found that MEC→CA1 Granger causality significantly decreased in APP-KI mice compared to that in WT (*p* = 2.1 × 10^–4^, Wilcoxon rank-sum test; Figure 5E), suggesting that the propagation of SWRs in the MEC→CA1 direction is impaired in APP-KI mice. By contrast, CA1cont Granger causality was comparable between APP-KI and WT mice (*p* = 0.080, Wilcoxon rank-sum test). The decreased MEC→CA1 Granger causality in APP-KI was also observed when BIC was used for the causality analysis (*p* = 0.0031, Wilcoxon rank-sum test), instead of Akaike information criterion. Together, these results indicated that MEC→CA1 coordination of SWRs was disrupted in APP-KI mice.

## Discussion

At the preclinical stage of APP-KI mice, we found (1) short-duration SWRs in the hippocampal CA1 and (2) impaired SWR coordination between the MEC and CA1. Fernandez-Ruiz and others previously showed in healthy brains that optogenetic prolongation of SWRs causally enhanced memory consolidation, indicating the critical role of long-duration SWRs in memory consolidation (Fernández-Ruiz et al., 2019). Long-duration SWRs would specifically provide higher spatial information from longer spike replays and larger number of neurons involved (Wilson and McNaughton, 1994; Foster and Wilson, 2006). Yamamoto and Tonegawa showed in healthy brains that long and multiplex SWRs in CA1 became disrupted when MEC input was optogenetically inhibited, demonstrating a critical role of SWRs in the MEC in supporting long-duration SWRs in the hippocampus (Yamamoto and Tonegawa, 2017). Although the mechanism of how MEC SWRs contribute to long-duration CA1 SWRs remains unknown, MEC inputs via the perforant pathway may modulate SWR generation in CA3 (Sullivan et al., 2011). Alternatively, MEC inputs by way of temporoammonic pathway may directly modulate SWRs in CA1. In either case, the disappearance of preceding SWRs in the MEC found in this study suggests that the MEC no longer contributes to the generation of long-duration hippocampal SWRs in APP-KI mice. The absence of long-duration SWRs may in turn cause memory consolidation abnormality at later stages of AD. Because SWR properties remained mostly intact in the MEC at the preclinical stage, it is likely that synaptic transmission from the MEC to the hippocampus (Dong et al., 2007; Palop et al., 2007), rather than SWR generation inside the MEC, becomes deteriorated and causes the SWR incoordination in APP-KI mice. Our finding of SWR incoordination at the preclinical stage points to a possible usage of entorhinal SWRs as an early biomarker for AD diagnosis.

## Data availability statement

The raw data supporting the conclusions of this article will be made available by the authors, without undue reservation.

## Ethics statement

The animal study was reviewed and approved by Institutional Animal Care and Use Committee at the University of California, Irvine.

## Author contributions

TF, HJ, and KI contributed to conception and design of the study. TS developed the APP-KI mouse line. HJ and KI conducted animal experiments and data acquisition and preprocessed the data. TF, SS, HJ, TS, AK, and KI contributed to methodology and software. TF, HJ, SS, and KI performed the analyses. All authors contributed to manuscript revision, read, and approved the submitted version.

## Supplementary material

Supplementary Figure 1 List of MEC recording sessions in WT and APP-KI mice.

Supplementary Figure 2 Histological validation of electrode positions in the MEC of WT and APP-KI mice.
