# Supplementary figures and images for: Impaired sharp-wave ripple coordination between the medial entorhinal cortex and hippocampal CA1 of knock-in model of Alzheimer’s disease
Source: Front Syst Neurosci. 2022 Aug 25;16:955178. doi: 10.3389/fnsys.2022.955178 (PMC9452631; doi:10.3389/fnsys.2022.955178)

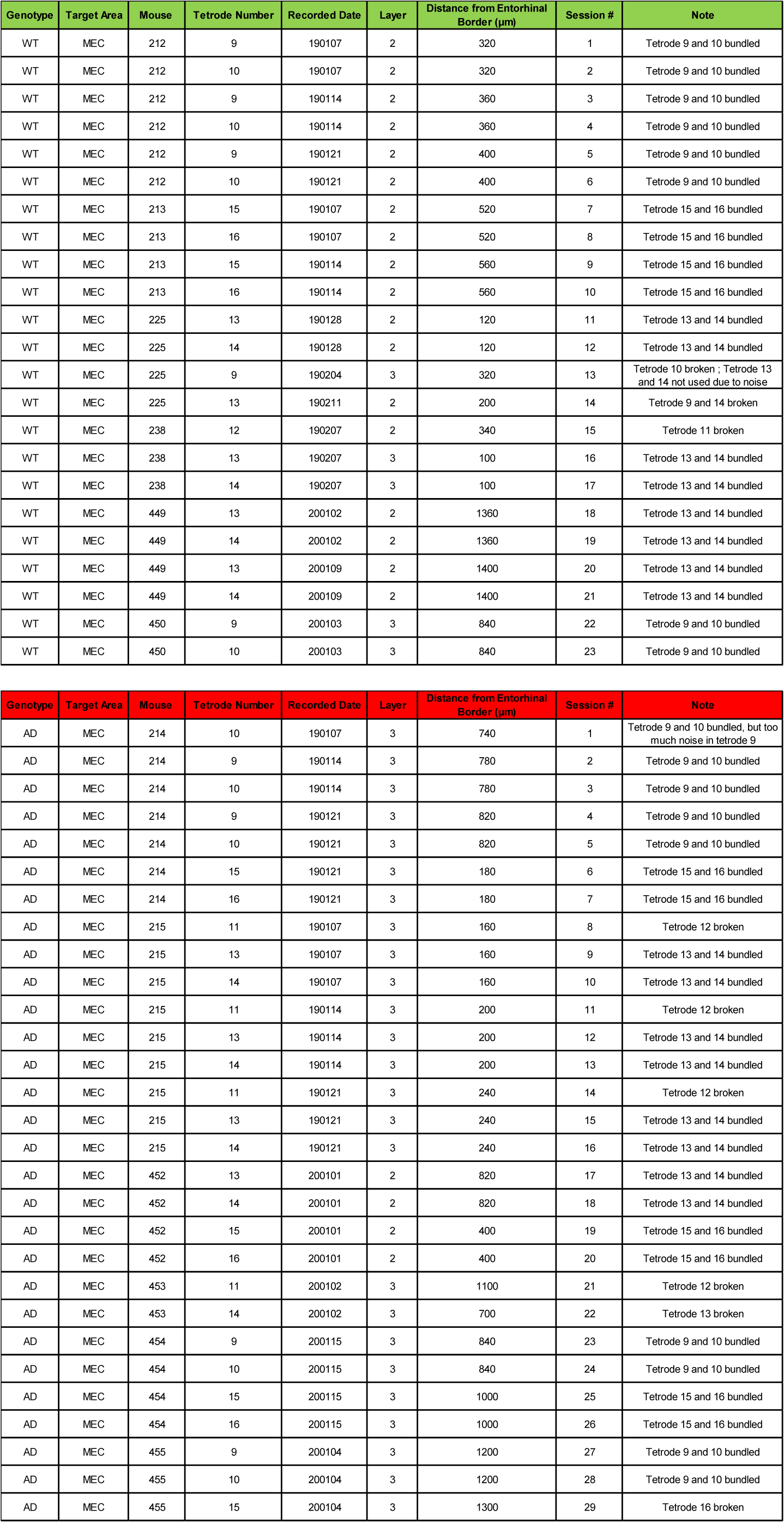

Supplement: Supplementary Figure 1 — List of MEC recording sessions in WT and APP-KI mice. [file Image_1.TIF]

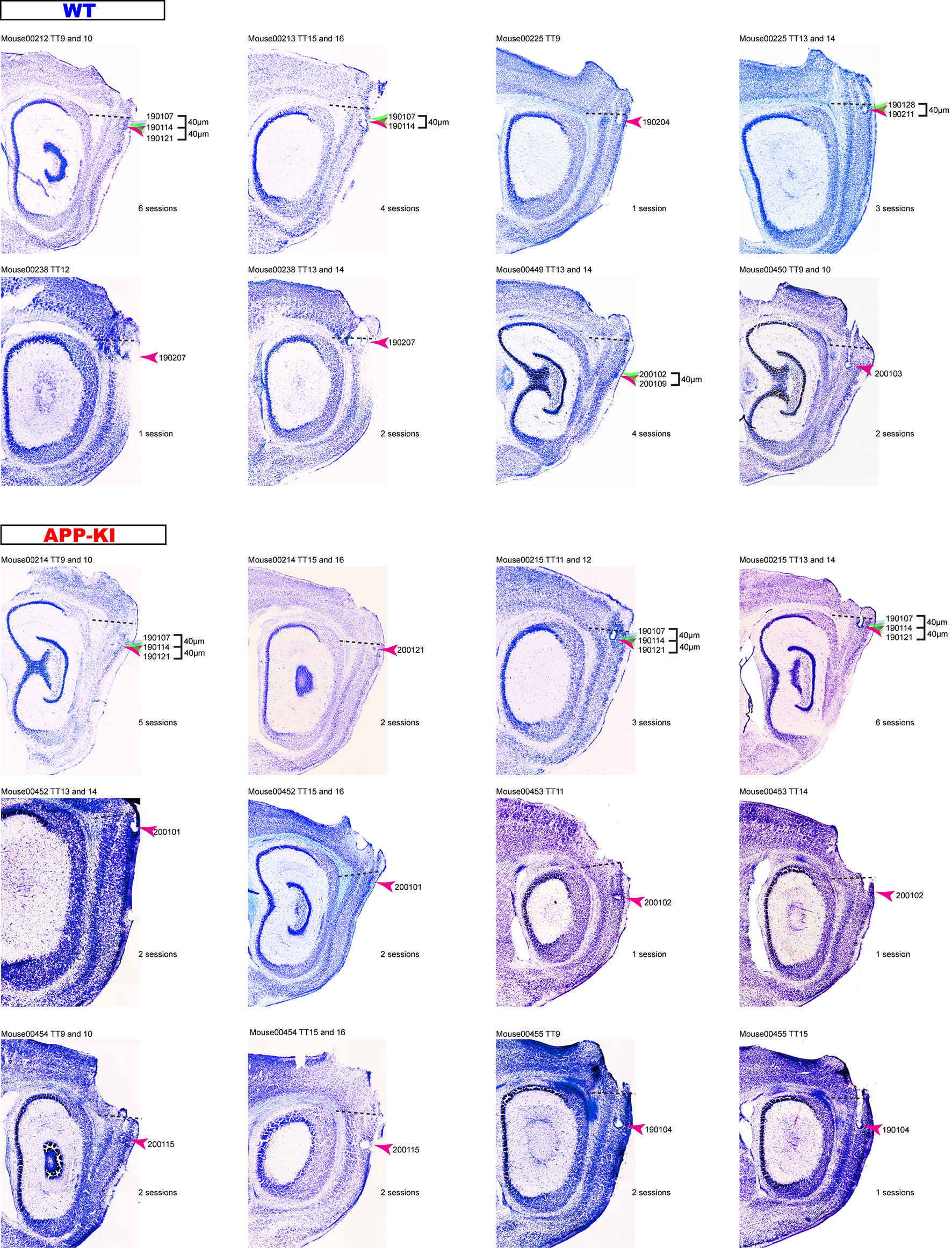

Supplement: Supplementary Figure 2 — Histological validation of electrode positions in the MEC of WT and APP-KI mice. [file Image_2.TIF]
